# Editorial: The potential role of melatonin in the regulation of abiotic stress in plants

**DOI:** 10.3389/fpls.2023.1271973

**Published:** 2023-09-12

**Authors:** Muhammad Ahsan Altaf, Milan Kumar Lal, Rahul Kumar Tiwari, Safina Naz, Vijay Gahlaut

**Affiliations:** ^1^ Key Laboratory for Quality Regulation of Tropical Horticultural Crops of Hainan Province, Sanya Nanfan Research Institute, Hainan University, Sanya, China; ^2^ ICAR-Central Potato Research Institute, Shimla, Himachal Pradesh, India; ^3^ Department of Horticulture, Bahauddin Zakariya University, Multan, Pakistan; ^4^ Department of Biotechnology and University Center for Research and Development, Chandigarh University, Mohali, India

**Keywords:** melatonin, abiotic stress management, photosynthesis, oxidative damage, root growth

Melatonin (N-acetyl-5-methoxytryptamine) is a dynamic molecule with a diverse range of functions in plants. Initially, melatonin was discovered in the bovine pineal gland in 1958. Melatonin was discovered in higher plantsn 1995 (Dubbels et al., 1995). Afterward, the multiple functions of melatonin have shown its significant promise in the field of plant physiology (Shi et al., 2017). Recently, melatonin has gained recognition as a versatile natural protector for crop plants, effectively mitigating both abiotic and biotic stresses (Tiwari et al., 2020; Altaf et al., 2021a). Melatonin plays a significant role as an antioxidant in plants, thereby enhancing their ability to withstand various stressors such as heat, drought, heavy metal exposure, salinity, chilling temperatures, as well as viral and fungal infections (Altaf et al., 2021a; Tiwari et al., 2021a; Tiwari et al., 2021b).

Furthermore, melatonin exerts its influence on plant physiology by regulating essential processes like rhizogenesis (the formation of lateral roots and root primordia), carbon assimilation, stomatal conductance, photochemical efficiency of photosystems, RuBisCO accumulation, chlorophyll molecule breakdown, and the ascorbate-glutathione (AsA-GSH) cycle in stressed plants (Moustafa-Farag et al., 2020; Sharma et al., 2020). The predominant mechanism through which melatonin exerts its anti-stress effects is enhancing the plant’s antioxidant defense mechanisms and aiding in scavenging ROS.

In the current scenario, climate change significantly influences agricultural productivity. Abiotic stress is the major agricultural constraint since it interferes with plant growth and yield (Hayat et al., 2023). Plants are exposed to various abiotic stresses, such as heavy metals, drought, temperature fluctuation, acid rain, salinity, and nutrient deficiency (Lal et al., 2023). Abiotic stresses significantly affect seed germination, flowering, root architecture, leaf photosynthesis, and seedling growth, ultimately reducing plant growth, yield, and quality (Rhaman et al., 2020). Therefore, it is crucial to have an area of research that includes plant physiological, morphological, and metabolic responses to determine the effect of abiotic stressors and identify possible defense mechanisms and mitigating approaches.

Application of plant growth regulators has become prevalent in agricultural crop production, enhancing yield quality and alleviating abiotic challenges’ impact (Altaf et al., 2023b). Melatonin regulates various plant physiological functions such as seed germination, photosynthetic efficiency, root architecture system, mineral nutrient uptake, maintained redox homeostasis, secondary metabolites production and balanced antioxidant enzymes system in plants (Altaf et al., 2021b). Compelling evidence shows that melatonin positively regulates the abiotic stress tolerance in plants. The majority of these studies propose that melatonin plays a dual role in plant defense mechanisms. Firstly, it appears to act as the frontline defense against reactive oxygen species (ROS), effectively neutralizing free radicals and preventing their harmful effects. Secondly, melatonin is involved in the second line of defense by regulating the expression of various genes that respond to stress conditions. This dual function positions melatonin as a crucial factor in helping plants cope with environmental challenges (Altaf et al., 2023a).

This Research Topic focuses on the potential role of melatonin in regulating abiotic stress in plants. We aim to ask whether and how melatonin regulates plant growth and stress responses and how the melatonin network interacts with other signalling pathways. This Research Topic contains seven original research and two review articles.

Zhao and Hu described the potential functions of melatonin in plants, including seed germination, leaf photosynthesis, root architecture system, redox homeostasis, antioxidant defence system, and seedling health index, as well as the influence of melatonin on plant growth and stress response. Melatonin acts as a growth regulator and bio-stimulator, enhancing plant tolerance to abiotic stress by improving nutrient uptake, osmolyte production, and cellular membrane stability. The review by Zhao and Hu provides valuable insights into how melatonin can be used to develop stress-tolerant horticultural crops in changing environments. Ahmad et al. highlighted melatonin’s interactions with nitric oxide and indole-3-acetic acid (IAA), which regulated physiological, morphological and metabolic functions. As a result of its endogenous application and regulatory roles involving NO and IAA, melatonin is capable of enhancing plant resilience and productivity under abiotic stresses.

The potential roles of melatonin were also revealed in different plant species under abiotic stress in this Research Topic. In common bean, Zhang et al. revealed that exogenous melatonin application significantly increased cell wall regulation pathway, plant growth, shoot length, and root morphological traits under salt stress. Melatonin treatment improved salt tolerance in over 65% of germplasm materials, and specific markers associated with cell wall synthesis enabled the prediction of melatonin-responsive varieties. This research suggests that melatonin can enhance salt tolerance in common bean by influencing the cell wall and providing markers for selecting stress-tolerant varieties. In barley, Jiang et al. found that rhizospheric melatonin supplementation favorably controlled the photosynthetic carbon assimilation and redox homeostasis in response to low temperatures. Similarly, exogenous melatonin reduced the impacts of drought stress in groundnuts by boosting endogenous melatonin concentration, which increased the antioxidant system and photosynthetic properties (Shreya et al.). Ghorbani et al. observed that melatonin application efficiently improved plant growth and biomass production, pigments content, proline production, antioxidant defence system, nitrogen metabolism, ion homeostasis, and reduced ROS production, electrolyte leakage and sodium accumulation in tomato seedlings under salinity stress. Awan et al. consistently noticed that melatonin markedly increased seed germination, biomass production, radical length, proline accumulation, and antioxidant enzymes system and reduced oxidative damage in soybean under salinity, heat and drought stress. In soybean, Jahan et al. found that spray treatment of melatonin considerably improved leaf gas exchange characteristics, enhancing water uptake efficiency, protected photosynthetic capacity, and balanced photosystems I and II and decreased ROS production under osmotic stress. Zulfiqar et al. revealed that preharvest melatonin treatment is viable for enhancing the cut tuberose’s postharvest quality. Various melatonin concentrations were tested, and all treatments significantly extended vase life by up to 4 days. Melatonin also improves various physiological characteristics, including increased levels of soluble proteins and sugars, enhanced catalase activity, and reduced oxidative stress markers, suggesting that melatonin preharvest application may be a useful tool for improving tuberose flower quality after harvest.

While this research presents promising results, further research is needed to address underlying questions. The focus of this study on melatonin’s role in regulating abiotic stress offers a new perspective and provides a basis for future research. It’s crucial to recognize that, beyond this specific Research Topic, we eagerly anticipate uncovering additional novel insights in the future regarding the potential role of melatonin in plant stress regulation. Abiotic stressors have a unique physiological response to melatonin treatments, so future research should investigate how these treatments can be optimized for various crop species. This will enable tailored strategies to enhance stress tolerance in diverse agricultural contexts.

## Author contributions

MA: Conceptualization, Writing – original draft, Writing – review & editing. ML: Conceptualization, Writing – review & editing, Writing – original draft. RT: Conceptualization, Writing – original draft, Writing – review & editing. SN: Writing – review & editing. VG: Writing – review & editing.
